# Shipi Shugan Decoction Protected against Sequela of Pelvic Inflammatory Disease via Inhibiting SIRT1/NLRP3 Signaling Pathway in Pelvic Inflammatory Disease Rats

**DOI:** 10.1155/2022/6382205

**Published:** 2022-09-05

**Authors:** Yan Wang, Yefang Huang, Ling Shi, Li Huang, Yi Wen, Yihong Cao, Zi Yang, Qian Liu, Xiaolan Yin, Xiaoli Ji

**Affiliations:** Department of Gynecology, Hospital of Chengdu University of Traditional Chinese Medicine, Chengdu 610072, China

## Abstract

Sequela of pelvic inflammatory disease (SPID) is a common and frequently occurring disease clinically. Traditional Chinese medicine (TCM) provided unique advantages in the treatment of SPID. In this study, we aimed to investigate the protective mechanism of Shipi Shugan Decoction (SSD), a Chinese herbal formula, on SPID using a SPID rat model. Mixed bacterial infection and mechanical injury were used for modeling. The chemical composition of SSD was analyzed by ultra-performance liquid chromatography-mass spectrometry (UPLC-MS). The inflammatory factors were detected by enzyme-linked immunosorbent assay (ELISA) and western blot techniques. We found that SSD dose-dependently inhibited the content of IL-18, IL-1*β*, TNF-*α*, and IL-6 in serum samples of SPID rats. The results from the hematoxylin and eosin (H&E) stain showed that SSD improved pathological injury of the uterus and fallopian tubes induced by a pathogen. In addition, SSD dose-dependently inhibited mitochondrial dysfunction and oxidative stress of SPID rats. The expression of SIRT1 was promoted, and NLRP3 inflammasome was deactivated by SSD gavage compared with the SPID group. Specifically, SIRT1 inhibitor EX-527 cotreatment significantly reversed the improvement effect of SSD on pelvic inflammatory disease in rats. Taken together, the results of this study suggest that Shipi Shugan Decoction may be an effective TCM for the treatment of SPID.

## 1. Introduction

Pelvic inflammatory disease (PID) is a common gynecological disease of the upper genital tract infection, including endometritis, salpingitis, ovarian cysts, pelvic peritonitis, and so on [1]. PID is usually caused by ascending infection of cervicovaginal microorganisms, of which the most important pathogens are *Neisseria gonorrhoeae* and *Chlamydia trachomatis* [2, 3]. Untreated PID can lead to a sequela of pelvic inflammatory disease (SPID). The clinical manifestations of the sequela of pelvic inflammatory disease (SPID) are chronic pelvic pain (CPP), infertility, pelvic adhesions, ectopic pregnancy, and recurrent inflammation [4]. Women with SPID suffer a lower quality of life in both physical health and psychological wellbeing. The pathological changes in SPID are tissue destruction, adhesions, hyperplasia, and scar formation. Antibiotics have a clear effect on the elimination of pathogens in the acute phase. However, they have little effect on the improvement of clinical symptoms in the chronic phase [5]. Traditional Chinese medicine (TCM) has certain characteristics and advantages in the treatment of PID [6, 7].

A Chinese herbal compound Man-Pen-Fang inhibited the inflammation reaction by promoting the apoptosis of inflammatory cells and downregulating the serum levels of inflammatory cytokines [8]. Chinese herbal medicine Radix Paeoniae Rubra could reduce the inflammatory symptoms of chronic PID by reducing the level of PTGS2 [9]. Furthermore, a meta-analysis suggested that Guizhi Fuling Wan decreased the score of traditional Chinese medicine symptoms and improved the therapeutic effect [10]. TCM believes that blood stasis is the basic pathogenesis for the occurrence and development of SPID [7, 11]. Pathogenic qi combating qi and blood, qi stagnation of liver, and yang insufficient of the kidney can lead to blood stasis. Blood stasis can obstruct Chong and Conception, and the uterus has a poor operation of qi and blood, eventually forming SPID [12, 13]. Thus, the principle of SPID TCM treatment is promoting blood circulation for warming and removing blood stasis, dispersing stagnated liver qi for relieving qi stagnation.

A Chinese herbal compound, Shipi Shugan Decoction (SSD), which is composed of Codonopsis Radix (Dangshen), Zingiber officinale Roscoe (Ganjiang), Macrocephalae Rhizoma (Baizhu), Glycyrrhizae Radix Et Rhizoma Praeparata Cum Melle (Zhigancao), Radix Bupleuri (Chaihu), Fructus Aurantii (Zhike), Paeoniae Radix Alba (Baishao), Coicis Semen (Yiyiren), Curcumae Rhizoma (Ezhu), and Astmgali Radix (Huangqi) as shown in Table 1 and Supplementary Figure 1A is effective in dispersing blood stasis and dredging collaterals. However, whether the SSD has an ameliorating effect on SPID has not yet been reported. The present study showed that the therapy of SSD had a significant effect on relieving SPID in a rat model. The effects of SSD on inflammation of the uterus and fallopian tubes, oxidative stress, and the histopathological alterations, as well as SIRT1/NLRP3 signaling pathway activity in the uterus, have been addressed.

## 2. Materials and Methods

### 2.1. Preparation of the Drug

The composition of SSD was shown in Table 1 and Supplementary Figure 1A. 10 kinds of TCM were soaked for 1 hour and decocted for 2 hours. After that, the juice was concentrated at 100 mL (110 g/100 mL). The clinically equivalent dose was used as the low-dose SSD. After the juice was concentrated 4 times, it was used as the high dose of SSD.

Fuke Qianjin Capsules, as positive control drugs, were purchased from Zhuzhou Qianjin Pharmaceutical Co., Ltd., (Z30020024; Zhuzhou, China). The equivalent dose for rats was 0.22 g/kg • day based on the calculation of animal dose and human equivalent dose for the drug [14].

### 2.2. Ultra-Performance Liquid Chromatography-Mass Spectrometry (UPLC-MS) Analysis

The chemical composition of SSD was analyzed by UPLC-MS. The UPLC-MS analysis was carried out with a hybrid Quadrupole-TOF LC/MS/MS Mass Spectrometer (B Sciex Instruments, Shimadzu LC30) using a Shimadzu InerSustain C18 column (2 *µ*m, 100 × 2.1 mm) with the column temperature at 35°C. The mobile phase was acetonitrile (A)-0.1% HCOOH–H2O (B) at a flow rate of 1 mL/min in gradient elution as follows: 0–4 min, 5% A; 4–12 min, 20% A; 12–14 min, 15% A; 14–26 min, 46% A; 26–28 min, 100% A; 28–30 min, 5% A. Electrospray ionization (ESI) positive and negative ion modes were used for detection. The ESI source conditions were set as follows: Ion Source Gas1 (Gas 1): 50, Ion Source Gas2 (Gas 2): 50, Curtain Gas (CUR): 25, Source Temperature: 500°C/450°C (positive ion/negative ion), Ion Sapary Voltage Floating (ISVF) 5500 V/4400V(positive ion/negative ion), TOF MS scan range: 100–1200 Da, product ion scan range: 50–1000 Da, TOF MS scan accumulation time 0.2 s, and product ion scan accumulation time 0.01 s. The secondary mass spectrum was obtained by information-dependent acquisition (IDA) with high sensitivity mode.

### 2.3. Animals Grouping and Experimental Design

80 Sprague-Dawley (SD) female rats (SPF grade, aged 12 weeks, body weight from 200 to 220 g) were purchased from Chengdu Dossy Experimental Animals CO., LTD. (Chengdu, Sichuan; SCXY (Chuan) 2020–034) and raised in the Hospital of Chengdu University of Traditional Chinese Medicine. The feeding environment was 21 to 23°C, with a relative humidity of 40%–60% and a 12 h light-dark cycle. SD rats are allowed to eat and drink freely. All rats were adaptively fed for 7 days before the experiments. All experiments were approved according to the Ethics Committee of the Hospital of Chengdu University of Traditional Chinese Medicine (2022DL-004).

For the first part of the experiment, 40 SD rats were divided into 5 groups using a random number table, with 8 rats in each group. 5 rats died (mortality rate 12.5%) in the process of the experiment probably due to low activity and poor diet, and 35 rats have remained. The number of rats used for follow-up experiments was as follows: sham group (*n* = 8), SPID group (*n* = 6), high-dose SSD group (*n* = 8), low-dose SSD group (*n* = 6), and Fuke Qianjin Capsules (FQC) group (*n* = 7). After the SPID model was established, the rats in the high-dose SSD group and low-dose SSD group were intragastrically administered with 6.64 g/kg • day and 1.66 g/kg • day SSD, respectively. The rats in the FQC group were intragastrically administered with 0.22 g/kg • day after the suspension of Fuke Qianjin Capsules. The rats in the sham group and SPID group were given the same amount of normal saline. For the second part of the experiment, 40 SD rats were divided into 5 groups using a random number table, with 8 rats in each group. 7 rats died (mortality rate 17.5%) in the process of the experiment probably due to low activity and poor diet, and 33 rats have remained. The number of rats used for follow-up experiments was as follows: sham group (*n* = 8), SPID group (*n* = 6), SSD group (*n* = 6), SIRT1 inhibitor (EX527) group (*n* = 6), and SSD + EX527 group (*n* = 7). After the SPID model was established, the rats in the EX527 group were intragastrically administered with a 5 mg/kg/day SIRT1 inhibitor (Sigma, Munich, Germany; No. E7034). The rats in SSD + EX527 group were intragastrically administered with 5 mg/kg/day and 6.64 g/kg • day SSD. The drug interventions in all rats were continued for 14 days. Then, all rats fasted for 12 h. Blood samples, uterus, and fallopian tube tissues were collected for analysis.

### 2.4. Rat SPID Model Construction

Mixed bacterial infection and mechanical injury were used for modeling. Rats were anesthetized by intraperitoneal injection of 40 mg/kg pentobarbital sodium. Regarding mechanical injury, the endometrial tissue was injured using a blunt needle-tipped syringe, which entered the uterine cavity and was pulled back and forth twice along the uterine wall. Regarding simultaneous bacterial infection, the mixed bacterial solution was composed of *Staphylococcus aureus* (*S. aureus*, No. 44152) and pathogenic *Escherichia coli* (*E. coli*, No. 26002). 0.1 mL (1 × 10^8^ CCU/mL; 1 : 1) of the bacterial solution was injected into the uterine cavity, and the rats were placed upside down for 3 minutes. The rats in the sham group were treated with the same volume of normal saline without damaging the endometrial tissue. The whole blood cell count in the rats after modeling was assessed every week. The SPID model was formed when the number of total leukocytes and total lymphocytes returned to normal.

### 2.5. Hematoxylin and Eosin (H&E) Stain

After fixation in 3% glutaraldehyde and 1% osmium tetroxide for 2 hours, uterus and fallopian tube tissues were used for H&E stain. Uterus and fallopian tube tissues were embedded in epoxy resins to prepare 5 *μ*m thick paraffin sections under electron microscopy. After deparaffinization with different concentrations of acetone, the sections were stained with hematoxylin and eosin. Six image area visual fields were randomly selected and assessed using a Panthera Upright Compound Microscope (Motic, Xiamen, China) under 100× and 400× magnifications.

### 2.6. Transmission Electron Microscopy (TEM)

5 *μ*m thick paraffin sections of the uterus and fallopian tube tissues were collected on a 300-mesh copper-mesh TEM grid. The sections were stained with uranyl acetate for 10∼15 min and lead citrate for 1∼2 min. A transmission electron microscope (JEM-1400FLASH, JEOL, Japan) was used to collect images of the TEM grid. Each TEM grid was observed and photographed at 6000× magnification to observe the specific lesions.

### 2.7. Oxidative Stress Biomarker Assay

The contents of glutathione peroxidase (GSH-Px, No. A005-1-2) in serum samples were detected by the colorimetry method using a microplate reader according to the kit instructions (Nanjing Jiancheng Bioengineering Institute, Jiangsu, China). Superoxide dismutase (SOD) content in serum samples was measured using the SOD assay Kit-WST1 (No. A001-3-2). Malondialdehyde (MDA) content in serum samples was detected by the method of thiobarbituric acid (TBA) according to the manufacturer's instructions (No. A003-1-2).

### 2.8. Mitochondrial Membrane Potential (ΔΨm) Assay

A mitochondrial-specific fluorescence probe JC-1 (Beyotime, Shanghai, China; No. C2006) was used to test ΔΨm. Briefly, uterine tissue sections were incubated with a mixture of JC-1 stain at 37°C for 20 min. A FACS Calibur Flow Cytometer (BD Biosciences) was used to analyze the fluorescence intensity of JC-1aggregates (red) and monomers (green).

### 2.9. Enzyme-Linked Immunosorbent Assay (ELISA)

The expressions of interleukin-18 (IL-18) (Abcam, USA, No. ab213909), IL-6 (Abcam, USA, No. ab234570), IL-1*β* (Abcam, USA, No. ab255730), tumor necrosis factor-*α* (TNF-*α*) (Abcam, USA, No. ab236712), and reactive oxygen species (ROS; BlueGene Biotech, Shanghai, China, No. 21175D-96) in serum samples were quantified according to the instructions of the ELISA kits. In brief, uterus and fallopian tube tissues were placed in precooled phosphate-buffered saline (PBS) and homogenized. The supernatant was collected by centrifugation at 4000 r/min for 10 min at 4°C. The BCA assay kit (Thermo Fisher Scientific, USA) was used to determine the protein content of the supernatant. 15 ng/*μ*L of the protein samples was used for incubation with corresponding antibodies. The absorbance was measured at 450 nm wavelength and was estimated using an enzyme-linked immune monitor (Thermo Fisher Scientific, USA). The concentration of these proteins in the sample was calculated from the standard curve.

### 2.10. Western Blot Analysis

The total proteins of the uterus and fallopian tube lyses solution were collected in RIPA buffer (Signaling Technology, Inc.). The protein concentration was determined using a BCA kit (Beyotime, Shanghai, China). Total protein (30 *µ*g/sample) was separated via 10% SDS-PAGE and to nitrocellulose membranes. Use 5% skimmed milk powder to block the membranes. The corresponding protein antibodies were as follows: silent information regulator 1 (SIRT1; Abcam, USA, No. ab110304; 1/1000), Nod-like receptor protein 3 (NLRP3; Abcam, USA, No. ab263899; 1/1000), caspase-1 (Abcam, USA, No. ab286125; 1/1000), and *β*-actin (Boster, Chian, No. BM0627; 1/1000). Then, the membrane washing was performed with Tris-buffered saline/0.1% Tween (TBST) and incubated for 1.5 hours with an HRP Goat anti-Rabbit IgG (Abcam, No. ab6721; 1/1000). The band visualization was carried out using the ECL system (Thermo Scientific, Rockford, IL, USA). *β*-actin was used as an internal control.

### 2.11. Statistical Analysis

The data were represented as means ± standard deviation (SD). Statistical analysis was performed with the SPSS software (version 19.0, SPSS Inc., Chicago, IL, USA). One-way analysis of variance (ANOVA) followed by Tukey's post hoc test and Student's unpaired *t*-test were used for comparison between groups. *P*-value <0.05 was considered statistically significant.

## 3. Results

### 3.1. Chemical Composition of SSD

We confirmed the chemical composition of the SSD using ultra-performance liquid chromatography-mass spectrometry (UPLC-MS). In the chromatographic profile of SSD, proline, 1,3-dimethylurate, anhydrodihydroartemisinin, prunin, falcarindiol, phthalic anhydride, and cinnamyl alcohol were detected in positive ionization scan mode (Supplementary Figure 1B), whereas D-(+)-Malic acid, 3,4-di-O-caffeoylquinic acid, paeoniflorin, naringenin, hesperidin, and soyasaponin Bb were detected in negative mode (Supplementary Figure 1C).

### 3.2. SSD Dose-Dependently Attenuated Inflammation of Uterine and Fallopian Tube in SPID Rats

**Figure 1:**
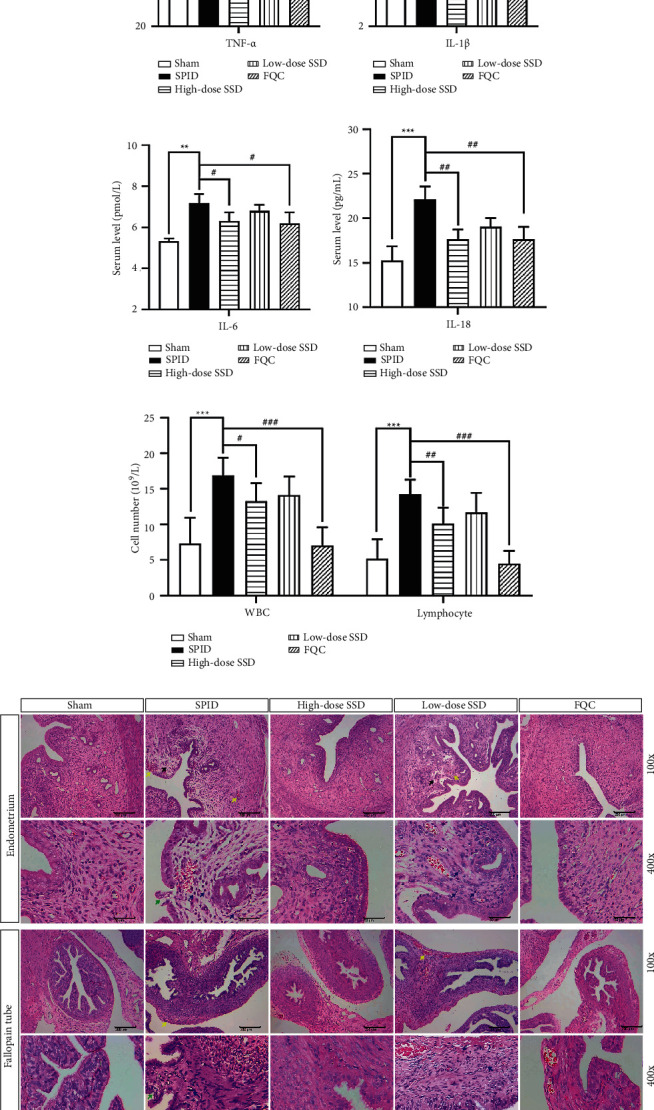
SSD dose-dependently attenuated inflammation of uterine and fallopian tube in SPID rats. 35 female rats were used for these experiments: Sham group (*n* = 8), SPID group (*n* = 6), high-dose SSD group (*n* = 8), low-dose SSD group (*n* = 6), and Fuke Qianjin Capsules (FQC) group (*n* = 7). After the SPID model was established, the rats in the high-dose SSD group and low-dose SSD group were intragastrically administered with 6.64 g/kg • day and 1.66 g/kg • day SSD, respectively. The rats in the FQC group were intragastrically administered with 0.22 g/kg • day after the suspension of FQC. The rats in the sham group and SPID group were given the same amount of normal saline. The contents of TNF-*α* (a) IL-1*β* (b) IL-6 (c) and IL-18 (d) in serum samples of rats in each group were tested by enzyme-linked immunosorbent assay (ELISA). (e) The numbers of WBCs and lymphocytes were counted under a microscope. (f) H&E stain was used to observe the pathological changes in the fallopian tube and uterine tissues. The magnification is 200× and 400×. Yellow arrow: vasodilation and hyperemia; green arrow: degeneration and necrosis of epithelial cells; blue arrow: lymphocyte; black arrow: endometrial lamina propria edema. H&E: hematoxylin and eosin; WBCs: white blood cells; TNF: tumor necrosis factor; IL: interleukin; SPID: sequela of pelvic inflammatory disease; SSD: Shipi Shugan Decoction; FQC: Fuke Qianjin Capsules. ^*∗*^*P* < 0.05 (versus sham), ^*∗∗*^*P* < 0.01 (versus sham), ^*∗∗∗*^*P* < 0.001 (versus sham), ^#^*P* < 0.05 (versus SPID), ^##^*P* < 0.01 (versus SPID), and ^###^*P* < 0.001 (versus SPID). Data represent the Mean ± SEM.

ELISA was performed to measure the levels of proinflammatory cytokines TNF-*α*, IL-6, IL-18, and IL-1*β* in serum samples. As the results showed in Figures 1(a)–1(d), SPID rats exhibited excessive levels of inflammation, mainly manifested in elevated TNF-*α*, IL-6, IL-18, and IL-1*β* expression levels compared with the sham group. SSD dose-dependently inhibited the content of TNF-*α*, IL-6, IL-18, and IL-1*β* in serum samples of SPID rats (Figures 1(a)–1(d)). After oral administration of the positive control drug FQC, the pathogen-induced productions of TNF-*α*, IL-6, IL-18, and IL-1*β* were reduced (Figures 1(a)–1(d)). Furthermore, H&E results were shown in Figures 1(e) and 1(f). In SPID group rats, the uterus and fallopian tube were characterized by obvious congestion and infiltrated by mass inflammatory cells, including white blood cells (WBCs) and lymphocytes. These results suggested that there was an inflammation response and lesion in the upper genital tract. Compared with the SPID group, SSD and positive control drug FQC significantly reduced the inflammatory cell infiltration in the upper genital tract (Figures 1(e) and 1(f)). These results indicated that there was an inflammatory response in SPID rats and SSD had anti-inflammatory effects.

### 3.3. SSD Dose-Dependently Inhibited Mitochondrial Dysfunction and Oxidative Stress of SPID Rats

**Figure 2:**
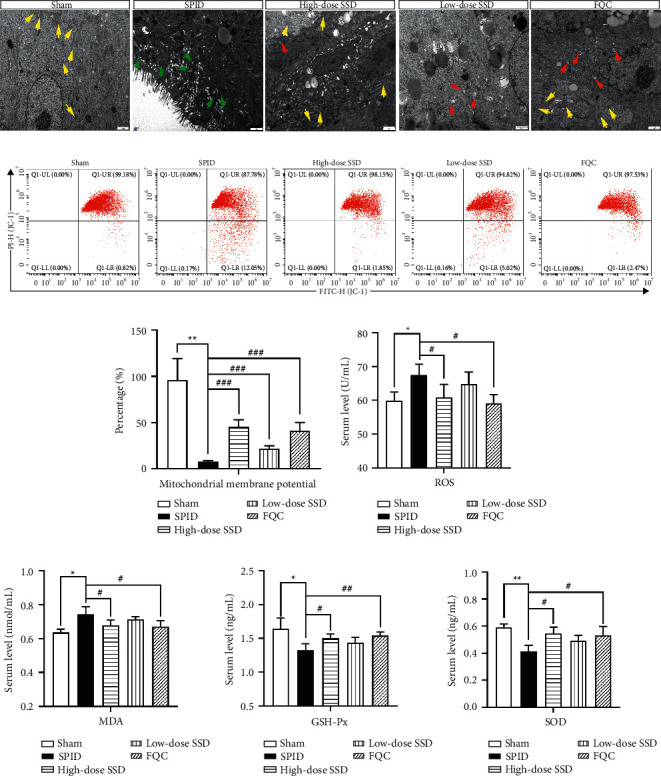
SSD dose-dependently inhibited mitochondrial dysfunction and oxidative stress of SPID rats. (a) The compromised mitochondria were tested by TEM. Scale, 1 *μ*m. Yellow arrow: normal mitochondria; green arrow: pyknotic mitochondria; red arrow: swollen mitochondria. (b and c) Flow cytometry was used to detect mitochondrial membrane potential. (d) The content of ROS in serum samples of rats in each group was tested by enzyme-linked immunosorbent assay (ELISA). The generation of MDA (e) GSH-Px (f) and SOD (g) in serum samples was analyzed by corresponding commercial kits. SPID: sequela of pelvic inflammatory disease; SSD: Shipi Shugan Decoction; FQC: Fuke Qianjin Capsules; TEM: transmission electron microscopy. ^*∗*^*P* < 0.05 (versus sham), ^*∗∗*^*P* < 0.01 (versus sham), ^#^*P* < 0.05 (versus SPID), ^##^*P* < 0.01 (versus SPID), and ^###^*P* < 0.001 (versus SPID). Data represent the mean ± SEM.

It is well documented that mitochondrial dysfunction can lead to a decline in energy production, generation of ROS, and induction of stress-induced apoptosis. Oxidative damage is a major cause leading to inflammation response. Thus, we studied the effect of SSD on mitochondrial oxidative stress of uterine tissues. The mixed bacterial solution treatment significantly promoted mitochondrial injury in uterine tissues, which was reversed by the positive control drug FQC and different concentrations of SSD (Figure 2(a)). Meanwhile, the ΔΨm was detected by JC-1 fluorescence stain. The decreased level of ΔΨm was observed in uterine tissues of SPID rats, and this increase was hindered by SSD administration in a dose-dependent manner (Figures 2(b) and 2(c)). We then confirmed that SSD and positive control drug FQC suppressed ROS and MDA expression, as well as increased GSH-Px and SOD expression compared with that in the SPID rat group (Figures 2(d)–2(g)). These results implied that the partial recovery of mitochondrial biogenesis was achieved by SSD administration.

### 3.4. Modulation of SSD on SIRT1/NLRP3 Signaling

**Figure 3:**
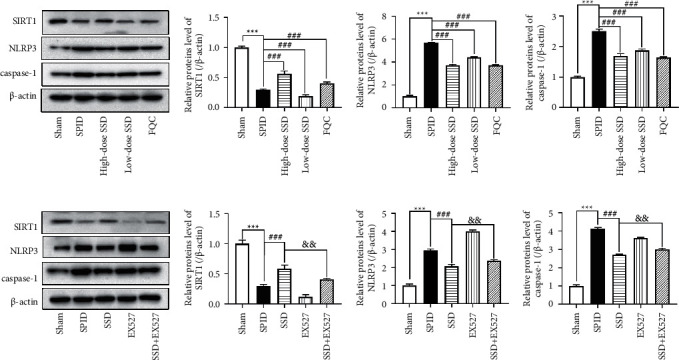
Modulation of SSD on SIRT1/NLRP3 signaling. (a and e) The protein expression levels of SIRT1, NLRP3, and caspase-1 were determined by Western blot analysis. β-actin was used as a loading control. (b, c, d, f, g, h) Quantitative analysis of western blot analysis normalized to SIRT1, NLRP3, and caspase-1.(versus sham),(versus SPID), and(versus SSD). Data represent the mean ± SEM.

Because SIRT1/NLRP3 signaling was the critical pathway in oxidative stress and inflammation induction, we explored the alteration of SIRT1/NLRP3 signaling pathway activity in SPID rat uterine tissues before and after TCM treatment. As shown in Figures 3(a) and 3(b), the SPID rat group showed a lower protein expression of SIRT1 than the sham group. After treatment with FQC and SSD, especially for a high-dose group of SSD and FQC, the protein expression of SIRT1 was increased in uterine tissues of SPID rats (Figures 3(a) and 3(b)). Subsequently, we measured the expression levels of NLRP3 inflammation-related proteins NLRP3 and caspase-1, which were evident differences between groups of sham and SPID model groups (Figures 3(a), 3(c), and 3(d)). After treatment with SSD, the expression of NLRP3 and caspase-1 was decreased in a dose-dependent manner (Figures 3(a), 3(c), and 3(d)). The data demonstrated that SSD stimulated SIRT1 and suppressed NLRP3 inflammasome activation in uterine tissues of SPID rats.

### 3.5. SSD against Inflammation and Pathological Damage of the Upper Genital Tract by Inhibiting SIRT1/NLRP3 Signaling

**Figure 4:**
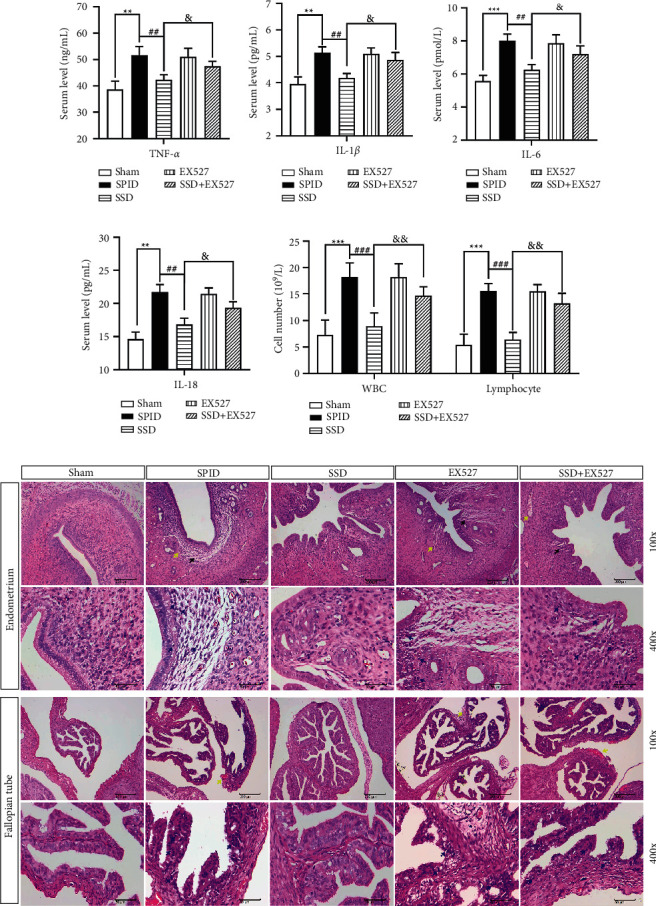
SSD against inflammation and pathological damage of upper genital tract by inhibiting SIRT1/NLRP3 signaling. 33 rats were used for these experiments: sham group (*n* = 8), SPID group (*n* = 6), SSD group (*n* = 6), SIRT1 inhibitor (EX527) group (*n* = 6), and SSD + EX527 group (*n* = 7). After the SPID model was established, the rats in the EX527 group were intragastrically administered with a 5 mg/kg/day SIRT1 inhibitor (Sigma, Munich, Germany; No. E7034). The rats in SSD + EX527 group were intragastrically administered with 5 mg/kg/day and 6.64 g/kg • day SSD. The rats in the sham group and SPID group were given the same amount of normal saline. The content of TNF-*α* (a) IL-1*β* (b) IL-6 (c) and IL-18 (d) in serum samples of rats in each group was detected by enzyme-linked immunosorbent assay (ELISA). (e) The numbers of WBCs and lymphocytes were counted under a microscope. (f) H&E stain was used to observe the pathological changes in the fallopian tube and uterine tissues. The magnification is 200× and 400×. Yellow arrow: vasodilation and hyperemia; blue arrow: lymphocyte; black arrow: endometrial lamina propria edema; H&E: hematoxylin and eosin; WBCs: white blood cells; TNF: tumor necrosis factor; IL: interleukin; SPID: sequela of pelvic inflammatory disease; SSD: Shipi Shugan Decoction; FQC: Fuke Qianjin Capsules. ^*∗∗*^*P* < 0.01 (versus sham), ^*∗∗∗*^*P* < 0.001 (versus sham), ^##^*P* < 0.01 (versus SPID), ^###^*P* < 0.001 (versus SPID), ^&^*P* < 0.05 (versus SSD), and ^&&&^*P* < 0.001 (versus SSD). Data represent the Mean ± SEM.

We then focused on whether the SIRT1/NLRP3 signaling mediates the anti-inflammatory and antipathological damage effects of SSD. The SIRT1-specific inhibitor EX527 was used to treat SPID rats, which decreased the protein expression of SIRT1 and increased the expression of NLRP3 and caspase-1 (Figures 3(e)–3(h)). SIRT1 inhibition resulted in increased levels of TNF-*α*, IL-6, IL-18, and IL-1*β* which were reduced by SSD treatment (Figures 4(a)–4(d)). In addition, the results of the H&E stain showed that EX527 treatment could block the ameliorative effect of SSD on endometrial tissue injury and inflammation reaction in the upper genital tract (Figures 4(e) and 4(f)). The data indicated that the activation of SIRT1/NLRP3 signaling reversely hindered the anti-inflammatory and antipathological damage effects of SSD on SPID rats.

### 3.6. SSD against Mitochondrial Dysfunction and Oxidative Stress by Inhibiting SIRT1/NLRP3 Signaling

**Figure 5:**
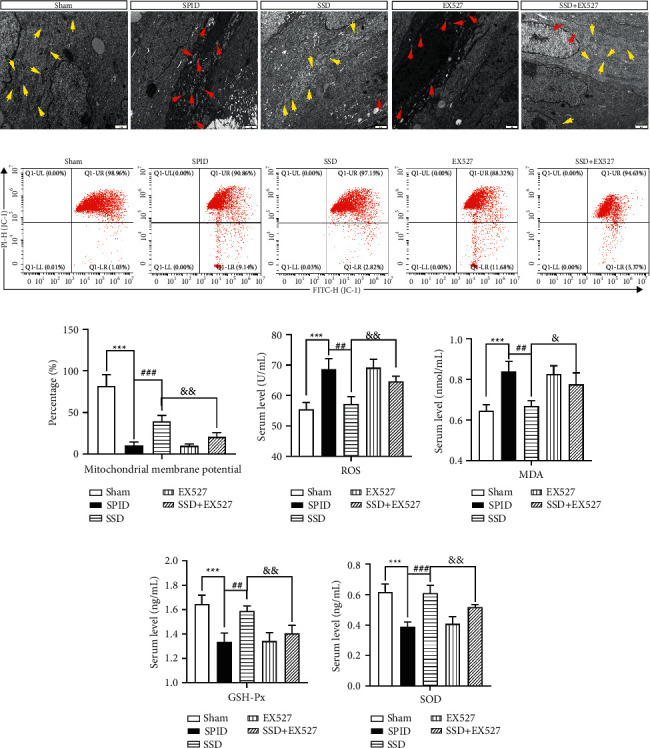
SSD against mitochondrial dysfunction and oxidative stress by inhibiting SIRT1/NLRP3 signaling. (a) The compromised mitochondria were measured using TEM. Scale, 1 *μ*m. Yellow arrow: normal mitochondria; red arrow: swollen mitochondria. (b and c) Flow cytometry was used to analyze mitochondrial membrane potential. (d) The content of ROS in serum samples of rats in each group was tested by enzyme-linked immunosorbent assay (ELISA). The generation of MDA (e) GSH-Px (f) and SOD (g) in serum samples was assayed by corresponding commercial kits. SPID: sequela of pelvic inflammatory disease; SSD: Shipi Shugan Decoction; FQC: Fuke Qianjin Capsules; TEM: transmission electron microscopy. ^*∗∗∗*^*P* < 0.001 (versus sham), ^##^*P* < 0.01 (versus SPID), ^###^*P* < 0.001 (versus SPID), ^&&^*P* < 0.05 (versus SSD), and ^&&^*P* < 0.01 (versus SSD). Data represent the Mean ± SEM.

TEM experiments showed that compromised mitochondria were redetected in the uterine tissue of SPID rats coprocessed with SSD and SIRT1 inhibitor EX527 (Figure 5(a)). Furthermore, we found that after SPID rats were treated with SIRT1 inhibitor EX527, a significant decrease in ΔΨm was observed in uterine tissue compared with the SSD-treated group (Figures 5(b) and 5(c)). The decreased levels of ROS and MDA, as well as increased levels of GSH-Px and SOD induced by SSD, were significantly reversed by SIRT1 inhibition (Figures 5(d)–5(g)). These results illustrated that SSD eliminated mitochondrial dysfunction and oxidative stress through stimulating SIRT1 expression and inhibiting the activity of the NLRP3 inflammasome.

## 4. Discussion

The results of this study indicated that SSD improved inflammatory damage of the upper genital tract and reduced mitochondrial oxidative stress in uterine tissues of SPID rats. The related mechanism is to stimulate the expression of SIRT1 and inhibit the activity of the NLRP3 inflammasome.

SSD included Codonopsis Radix, ginger (Zingiber officinale Roscoe), Macrocephalae Rhizoma, Glycyrrhizae Radix Et Rhizoma Praeparata Cum Melle, Radix Bupleuri, Fructus Aurantii, Paeoniae Radix Alba, Coicis Semen, Curcumae Rhizoma, and Astmgali Radix. Each of them has been extensively used in the treatment of inflammatory diseases. Codonopsis Radix was rich in various chemical constituents, including terpenoids, saponins, alkaloids, and phenolic compounds with anti-inflammatory and immunomodulatory properties [15]. The bioactive constituents of ginger contained terpenes, polysaccharides, lipids, and organic acids, which had antioxidant, anti-inflammatory, and antimicrobial effects [16]. Another previous study reported that the application of Macrocephalae Rhizoma and Astmgali Radix inhibited the generation of proinflammatory cytokines in the lipopolysaccharide (LPS)-induced chronic inflammation model [17]. Moreover, clinical trials have shown that glycyrrhetinic acid and glycyrrhizic acid from Glycyrrhizae Radix Et Rhizoma Praeparata Cum Melle were effective against inflammatory intestinal diseases [18]. Radix Bupleuri, Fructus Aurantii, and Paeoniae Radix Alba have been demonstrated to reduce the inflammatory factors and increase anti-inflammatory factors in the serum of depressive-like rats [19]. Coicis Semen was applied for treatment with ischemia-reperfusion injury, and it was proved to inhibit oxidative stress and promote angiogenesis [20]. Notably, TCM compounds with Curcumae Rhizoma, Astmgali Radix, Glycyrrhizae Radix Et Rhizoma Praeparata Cum Melle, and Radix Bupleuri as the core medicines had significant effects on patients with CPP caused by SPID [21]. In our study, we determined that SSD suppressed the generation of proinflammatory factors in serums and inflammatory cell infiltration in the uterus and fallopian tubes of SPID rats, implying that SSD could effectively eliminate the pelvic inflammatory disease in in vivo study.

Mitochondrial dysfunction and oxidative stress injury were important pathophysiological mechanisms of PID [22, 23]. It was reported that the reduced activity of mitochondrial respiratory chain complex 1 could promote ROS accumulation, leading to depolarization of mitochondrial membrane potential and changes in membrane permeability [24, 25]. Superoxide radicals, hydrogen peroxide, hydroxyl radicals, and so on are collectively referred to as ROS [26, 27]. The excessive release of ROS induced cell damage and changed cell function by regulating protein activities and gene expression. A large amount of oxidative stress product MDA was produced when the local inflammation waterfall was formed in the SPID rat model, which in turn induced oxidative stress injury [28]. SOD and GSH-Px, a class of antioxidant enzymes, had the effect of scavenging oxygen-free radicals [29, 30]. In the present study, the results show that SSD concentration dependently protested mitochondrial dysfunction, exhibiting similar efficacy to positive drug. Additionally, SSD enhanced SOD and GSH-Px levels and reduced MDA and ROS production in serum samples of SPID rats.

SIRT1 was a nicotinamide adenine dinucleotide (NAD+)-dependent deacetylase that participated in a variety of physiological and pathological processes and played an important role in inflammatory diseases [31, 32]. The study has shown that SIRT1 can participate in the regulation of cell activities such as inflammation, oxidative stress, and mitochondrial function [33, 34, 35]. Specifically, SIRT1 inhibited the occurrence and development of inflammation by regulating the expression of NLRP3 [36, 37]. NLRP3, apoptosis-associated speck-like protein (ASC), and pro-caspase-1 made up the NLRP3 inflammasome, subsequently activating caspase-1. Activated caspase-1 further catalyzed the secretion of proinflammatory cytokines IL-1*β* and IL-18 and finally induced inflammatory responses and impairments [38, 39, 40]. A review revealed that the expression of NLRP3 inflammasome was increased in the endometrium of women with unexplained recurrent pregnancy loss (RPL) [41]. A previous study found that *Gardnerella vaginalis* induced the expression of NLRP3 inflammasome-dependent cytokines IL-1*β*, IL-18, as well as TNF-*α* in inflammation of the genital tract [42]. Melatonin improved mouse endometritis by constraining the level of NLRP3 activation [43]. Meanwhile, melatonin attenuated pelvic pain caused by inflammation of the prostate by downregulating SIRT1-dependent inhibition of the NLRP3 inflammasome. In the present study, we found that SSD exerted a protective effect on pelvic inflammatory disease through activation of the SIRT1 signaling and elimination of the NLRP3 inflammasome. Our results were similar to previous reports that various components of SSD have significant inhibitory effects on SIRT1/NLRP3 signaling activity. 6-Gingerol, a phenolic compound extracted from ginger, significantly suppressed autophagy-induced NLRP3 inflammasome and neuronal apoptosis [44]. The fermented Chinese formula Shuan-Tong-Ling, including Codonopsis Radix, Macrocephalae Rhizoma, Radix Bupleuri, Paeoniae Radix Alba, Astmgali Radix, and so on, protected against cerebral ischemia/reperfusion injury by reducing inflammation and apoptosis through activation of the SIRT1 signaling pathway [45]. The compounds from Fructus Aurantii significantly inhibited liver inflammation in liver fibrosis mice by reducing NLRP3 expression [46]. Coixol, a plant polyphenol extracted from Coicis Semen, exerted anti-inflammatory effects on LPS-induced macrophage cells and diminished NLRP3 inflammasome activation [47]. In this study, the components of SDD synergistically improved the development of SPID by activating SIRT1 and inhibiting NLRP 3 inflammasome.

## 5. Conclusions

Our findings suggest that SSD reduced proinflammatory factor levels in serum and inhibited inflammation injury of the uterine and fallopian tube in SPID rats. Additionally, SSD inhibited mitochondrial dysfunction, and oxidative stress, promoted SIRT1 activation, and caused downregulation in the protein expression of NLRP3 inflammasome. Importantly, SIRT1 inhibitor EX527 significantly reversed the protective effect of SSD on SPID. Our results may provide new therapeutic approaches against SPID. Further research is needed to validate the clinical effectiveness of SSD.

## Figures and Tables

**Table 1:** The composition of Shipi Shugan Decoction (SSD).

Scientific name	Chinese name	Weight (g)	%
Codonopsis Radix	Dangshen	10.00	9.09
Zingiber Officinale Roscoe	Ganjiang	10.00	9.09
Macrocephalae Rhizoma	Baizhu	10.00	9.09
Glycyrrhizae Radix Et Rhizoma Praeparata Cum Melle	Zhigancao	5.00	4.55
Radix Bupleuri	Chaihu	10.00	9.09
Fructus Aurantii	Zhike	10.00	9.09
Paeoniae Radix Alba	Baishao	10.00	9.09
Coicis Semen	Yiyiren	10.00	9.09
Curcumae Rhizoma	Ezhu	15.00	13.64
Astmgali Radix	Huangqi	20.00	18.18
Total amount		110.00	100.00

## Data Availability

The datasets used or analyzed during the current study are available from the corresponding author upon reasonable request.

## Supplementary Materials

Supplementary Materials Supplementary Figure 1: chemical composition of SSD. (A) The composition of SSD. (B, C) The total ion chromatograms of SSD by UPLC-MS technique. (B) Chromatogram in positive ionization mode. (C) Chromatogram in negative ionization mode. SSD: Shipi Shugan Decoction; UPLC-MS: ultra-performance liquid chromatography-mass spectrometry.
