# Supplementary material for: Shipi Shugan Decoction Protected against Sequela of Pelvic Inflammatory Disease via Inhibiting SIRT1/NLRP3 Signaling Pathway in Pelvic Inflammatory Disease Rats
Source: Evid Based Complement Alternat Med. 2022 Sep 5;2022:6382205. doi: 10.1155/2022/6382205 (PMC9467799; doi:10.1155/2022/6382205)
Supplement: Supplementary Materials — Supplementary Figure 1: chemical composition of SSD. (A) The composition of SSD. (B, C) The total ion chromatograms of SSD by UPLC-MS technique. (B) Chromatogram in positive ionization mode. (C) Chromatogram in negative ionization mode. SSD: Shipi Shugan Decoction; UPLC-MS: ultra-performance liquid chromatography-mass spectrometry. [file 6382205.f1.pdf]

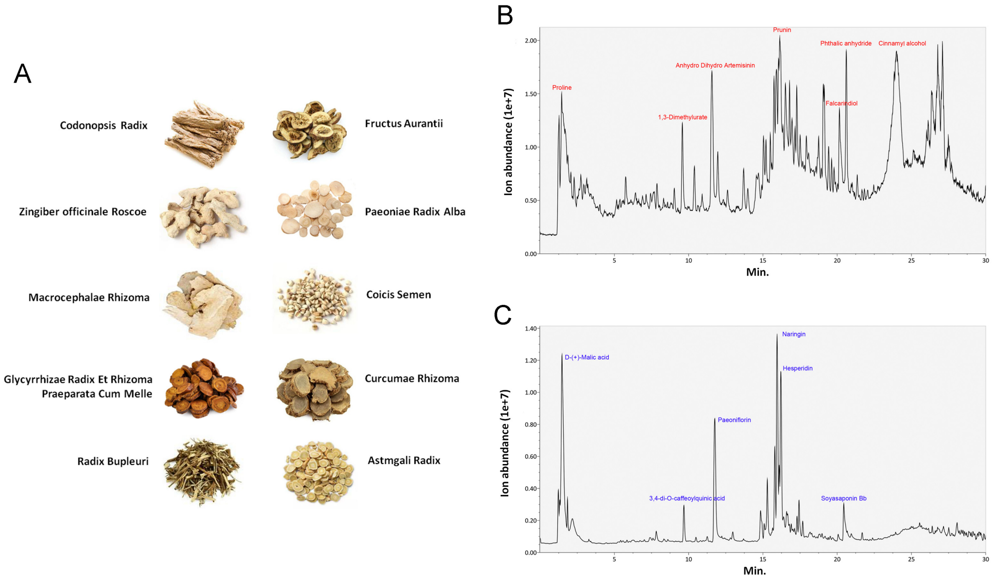
A
Codonopsis Radix
Fructus Aurantii
Zingiber officinale Roscoe
Paeoniae Radix Alba
Macrocephalae Rhizoma
Coicis Semen
Glycyrrhizae Radix Et Rhizoma Praeparata Cum Melle
Curcumae Rhizoma
Radix Bupleuri
Astmgali Radix
B
Ion abundance (1e+7)
Min.
Proline
1,3-Dimethylurate
Anhydro Dihydro Artemisinin
Prunin
Phthalic anhydride
Cinnamyl alcohol
Falcarindiol
C
Ion abundance (1e+7)
Min.
D-(+)-Malic acid
3,4-di-O-caffeoylquinic acid
Paeoniflorin
Naringin
Hesperidin
Soyasaponin Bb
